# Toward the commercialization of recombinant pharmaceuticals expressed in plants

**DOI:** 10.5511/plantbiotechnology.24.1130a

**Published:** 2025-03-25

**Authors:** Haruhiko Washida, Kyoji Yoshinaka, Okuto Yamada, Shoichiro Ookawa, Masayuki Yuki

**Affiliations:** 1 Research and Development Division, UniBio Corporation, 88, Niidagata, Nishikan-ku, Niigata 959-0597, Japan

**Keywords:** *Nicotiana benthamiana*, pharmaceutical protein, recombinant protein, transient expression, xenobiotics

## Abstract

Recent developments have shown that the production of recombinant proteins in plants is more useful than in microbial, insect, or mammalian cell-based expression systems in terms of cost-effectiveness, scalability, safety, and sustainability. Furthermore, transient expression systems in plants may be superior to stable transgenic plants in terms of cost, yield, environmental impact, and regulation compliance. Recombinant proteins, such as enzymes, growth factors, scaffolds, and antibodies are in high demand for use in the food and chemical industries, and will be in even greater demand for diagnostic, therapeutic, and pharmaceutical applications that require high-quality proteins. In this review, we summarize the comparison of recombinant protein expression strategies in mammalian cells, microorganisms, insects, and plants. Furthermore, the efficacy of protein expression in plant cultivation environments, the optimal protein extraction, purification methods, and costs and risks are discussed. We should be aware that the production of recombinant proteins has not only scientific challenges, but also economic and political issues that must be overcome.

## Introduction

The development of genetic engineering technology has made it possible to express useful proteins in heterologous organisms, not only for research purposes, such as exploring the function of substances, but also for therapeutic purposes, such as insulin. Such proteins, typically called “recombinant proteins”, has already been put to commercial use (Itakura et al. 1977). Five hundred and forty-one biopharmaceuticals, containing 435 distinct ingredients, such as enzymes, coagulation and fibrinolysis system factors, hormones, vaccines, interferons, erythropoietin, cytokines, toxins, antibodies, and antibody-drug complexes, were recently approved in the United States and European Union as of the end of June 2022 (Walsh and Walsh 2022). However, almost 100 products were withdrawn for commercial reasons (Walsh and Walsh 2022).

Many applications of recombinant protein technologies in plants have been developed, such as rice expressing two β-carotene biosynthesis genes in the endosperm to compensate for vitamin A deficiency (Ye et al. 2000), genetically modified strawberries producing interferon as a treatment for periodontal disease in dogs (Matsumura and Tabayashi 2022), and expression of β-glucocerebrosidase as a Gaucher disease type I enzyme replacement therapy in carrot cultured cells (Shaaltiel et al. 2007). These were produced by transgenic crops, but other methods utilize transient expression systems. This strategy enables the production of recombinant proteins in a relatively short time (1 or 2 weeks) after gene transfer, which can facilitate the creation of novel medical proteins such as vaccines, antibody drugs, and other similar products and clinical development (Marsian and Lomonossoff 2016).

## Recombinant proteins

Recombinant proteins are important tools not only for studying biological processes but also for cosmetic, therapeutic, and medical applications, and generating a recombinant protein requires the use of an expression system. To select the appropriate host, in addition to an ease of protein expression for each host, the long-term economics, including the safety, scalability, production scale adjustment capability, and the time to market, must be considered in order to determine the advantages and disadvantages (Table 1).

**Table 1:** Table 1. Comparison of different expression hosts. Edited from Ayyar et al. (2017).

Characteristic	Transient plant expression	Transgenic plants	*E. coli*	Yeast	Insect cells	Mammalian cells	Transgenic animals	Cell free
Safety	High	High	Low	Moderate	Moderate	Moderate	High	Depends on origin
Production scale adjustment capability	Very easy	Very easy	Very easy	Very easy	Moderate	Moderate	Unknown	Unknown
Yield rate	High	High	Medium	High	Medium-High	Medium-High	High	High
Time to market	Low	High	Low	Low	Moderate	Moderate	Very high	Unknown
Genetic manipulation	Easy	Hard	Easy	Moderate	Hard	Hard	Hard	Easy
Folding	Correct folding	Correct folding	Generally refolding required	Refolding may be required	Correct folding	Correct folding	Correct folding	Refolding may be required
Glycosylation	Present	Present	Absent	Present	Present	Present	Present	Absent
Posttranslational modifications	Present	Present	Absent	Present	Present	Present	Present	Absent

Table 1 compares the advantages and disadvantages of recombinant protein expression technologies. The *Escherichia coli* (*E. coli*) system is the easiest way to express proteins inexpensively and rapidly, but it lacks many of the post-translational modifications found in eukaryotes. The ability of *E. coli* in protein folding and disulfide bond formation is not sufficient for the majority of recombinant proteins, and bacteria pose an endotoxin risk. Hence, in order to overcome this limitation of *E. coli*, alternative expression systems that utilize eukaryotic organisms can be employed (Table 1).

In contrast to *E. coli*, the eukaryotic yeast, insect, mammalian, and plant systems promote good protein folding and many post-translational modifications (Table 1). One of the best strategies for post-translational modification, including protein folding, is protein expression in mammalian cells, including human cells; HEK293 (Human Embryonic Kidney cells 293) and CHO (Chinese hamster ovary cells) are commonly available. HEK293 is a cell line established by transfecting human fetal kidney cells with DNA from human adenovirus type 5 (Graham et al. 1977). However, there is a risk of zoonosis in mammalian cells and extra costs regarding countermeasures. Viral contamination was discovered in Genzyme’s Gauche disease treatment drug plant that uses CHO cells (Bethencourt 2009). Furthermore, it was reported that one clinical trial subject treated with a Gauche disease drug expressed from cultured human cells has developed cancer (Holubar et al. 2018). In addition, there are ethical concerns with the use of human cells. HEK293 cells are derived from cells of aborted human fetuses, and although they were done legally, it is not known who the parents are or the reason for the abortion (van der Eb 2001), potentially resulting in ethical issues that may make them unusable in the future (Wong 2006).

In the case of insect cells, the baculovirus that is used to genetically modify insects and was recently used in influenza vaccine (Flublok®) production (Cox et al. 2008, 2015) is known to infect mammals (Ghosh et al. 2002). Therefore, its safety may be questionable.

Another option for high-throughput rapid expression of functional recombinant proteins is cell-free expression systems that utilize prokaryotic or eukaryotic transcription and translational machinery (Ojima-Kato et al. 2015, 2018). However, the efficacy of these systems for large-scale expressions is still unknown.

## Plant recombinant protein expression

The plant expression system maintains high standards in terms of safety, production scale adjustment capability, cost (yield rate), development time required for commercialization (time to market), and other items when compared with other recombinant protein production methods (Table 1). One concern that has developed in the plant expression system is that the glycosylation process is different between animals and plants, and special sugar residues in the host plant may cause allergic reactions (Bardor et al. 2003; Gomord et al. 2005). To overcome this concern, modification of plant glycosylation was demonstrated by the use of mutants (Sariyatun et al. 2021), the gene knockdown (Schähs et al. 2007; Shin et al. 2017; Uthailak et al. 2021, 2022), the gene knockout (Jansing and Bortesi 2022; Jansing et al. 2019), and the introduction of human glycosylation genes (Fujiyama et al. 2007; Strasser et al. 2008).

However, the former Medicago anti-influenza vaccine and anti-SARS-CoV2 vaccine COVIFENZ®, which are transiently expressed in *Nicotiana benthamiana*, had both been shown to be safe in clinical trials despite the host plant-derived glycosylation (Hager et al. 2022; Ward et al. 2021), and subsequently Health Canada has granted regulatory approval for COVIFENZ®. The results suggest that in the case of Medicago, differences between plant and mammalian glycans may not affect safety, but drug safety must be rigorously evaluated on a case-by-case basis.

The transgenic plant system is thought to be one of the best methods for producing recombinant protein. Once the recombinant protein gene is induced and genetically fixed, not only the seeds but also the plantlet produced by the tissue culture can be propagated easily, and then it should be possible to obtain the recombinant protein more easily by growing the plant on a semi-permanent basis. There are two plant-made veterinary biopharmaceuticals currently available on the market (Interberry α® and HERBAVAC™), expressed from strawberry receptacle and *Nicotiana benthamiana*, respectively. Human collagen was expressed from transgenic tobacco (Shoseyov et al. 2014). Human and mouse cytokines expressed in rice seeds (Fujiwara et al. 2010, 2016) are available on the market as research-use-only reagents from Oriental Yeast Corporation. One concern is that it takes time to grow to harvest. It should be noted that in some countries, cultivation in outdoor fields of GMOs requires attention because public consensus may not be reached (Blancke et al. 2015).

Plant transient expression may overcome the above concerns and may be an excellent and sophisticated protein production system since it is possible for short-term cultivation in closed greenhouses, and there would be less concern about the spread of seeds and other materials to the outside. Table 2 shows examples of commercial-use recombinant proteins produced by private companies. The other studies include the oral cholera vaccine (Baldauf et al. 2017; Hamorsky et al. 2013), malaria vaccine (Jones et al. 2013), influenza virus vaccine (Le Mauff et al. 2015; Shoji et al. 2009; Yusibov et al. 2011), zika virus vaccine (Shin et al. 2023), anti-Ebola virus monoclonal antibody (Olinger et al. 2012; Zeitlin et al. 2011), anti-West Nile virus monoclonal antibody (Lai et al. 2014), and anti-human programmed cell death 1 monoclonal antibody (Phakham et al. 2021). The anti-Ebola virus monoclonal antibody (ZMapp™) licensed by Mapp Biopharmaceutical (https://mappbio.com) was first tested in humans, and in a randomized controlled trial of ZMapp™, the estimated effect of that appeared to be beneficial but did not reach the pre-specified statistical threshold for efficacy (PREVAIL II Writing Group; Multi-National PREVAIL II Study Team 2016).

**Table 2:** Table 2. Examples of plant recombinant proteins produced in private companies using transient expression technologies (in alphabetical order).

Company	Country	Product	URL	ClinicalTrials.gov ID
				Clinical Trial Phase
Agrenvec	Spain	Collagen, Cytokines (research use only)	https://www.agrenvec.es/	NA
Baiya Phytopharm	Thailand	Vaccine (COVID-19)	https://baiyaphytopharm.com/	NCT05197712
				I Complete
Icon Genetics GmbH	Germany	Vaccine (Norovirus)	https://www.icongenetics.com/	NCT05508178
				I Complete
KBio	USA	ZMapp™ Antibody (Ebola virus)	https://kbio.com/	NCT02363322
				I Complete
Leaf Expression Systems	UK	Antibody, etc.	https://www.leafexpressionsystems.com/	NA
Mapp Biopharmaceutical	USA	ZMapp™ Antibody (Ebola virus)	https://mappbio.com/	NCT02363322
				I Complete
Medicago*	Canada	COVIFENZ® Vaccine (COVID-19)	NA	NCT04636697
				III Complete
UniBio	Japan	Epidermal growth factor (cosmetic material)	https://unibio-jp.com/en/	NA

*Aramis Biotechnologies (https://aramisbiotechnologies.com/en/) acquired assets and intellectual property of Medicago (Government of Canada 2023).

The major concerns are efficient gene transfer and expression to produce the recombinant proteins efficiently, but transient expression is more efficient than stable expression. Agroinfiltration of plant virus-based expression vectors in transient gene expression systems has been summarized recently (Nosaki et al. 2021a). The tobacco mosaic virus-derived magnICON® (Marillonnet et al. 2005) and TRBO vector (Lindbo 2007) systems, BeYDV-derived deconstructed vector (Moon et al. 2014), geminiviral replication systems (Rizvi et al. 2015), the Tsukuba system (Yamamoto et al. 2018), and T-DNA-based vectors (Nagaya et al. 2010; Yamasaki et al. 2018) are well known.

Viruses can express more proteins since they can multiply, migrate, and spread to tissues other than the inoculated leaf, but are limited by the size and number of transgenes. In contrast, *Agrobacterium*-based transient expression is relatively flexible in the size and number of transgenes but may be limited in the amount of expression. In *Agrobacterium*-mediated transformation, it may also be related to the fact that only one or a few T-DNAs are transferred and incorporated into the genome (Hiei et al. 1994). *Agrobacterium* cannot express the protein in cells other than those within the inoculated tissue. Viral systems seem to be a good method for plant transient expression, but what about cost considerations? Japanese law typically regulates the use of agroinfiltration under the less stringent GILSP (Good Industrial Large-Scale Practice) category, whereas the use of plant viral vectors is regulated under the more stringent Category 1 (e-Gov 2022) for the reason that viral vectors require more attention in containment, although the actual categorization may vary on a case-by-case basis.

Furthermore, for efficient recombinant protein production, the following improvements in plant growing conditions have been reported. The transient recombinant protein overexpression in plants can sometimes cause hypersensitive response necrosis since protein overexpression causes intracellular misfolding and unfolding during protein conformation, resulting in ER stress in the cell and production of reactive oxygen species (ROS), which in turn causes biological damage (Nosaki et al. 2021b). To inhibit its oxidation, Nosaki et al. (2021b) reported that spraying the leaves with the antioxidant ascorbic acid inhibited necrosis. Although there were reports of adding ascorbic acid during *Agrobacterium* infection to promote increased expression (Deguchi et al. 2020; Norkunas et al. 2018), applying the antioxidant ascorbic acid spray may be a simple and inexpensive challenge for overcoming difficult-to-express proteins in transient expression in plants.

In the relationship between the plant growing environment and recombinant protein expression, Matsuda and colleagues have reported changes in recombinant protein production by controlling the growing environment of plants before and after gene transfer. They have shown that the production of influenza hemagglutinin (HA) protein was enhanced by reducing the cultivation temperature from 25°C to 20°C (Matsuda et al. 2017a) and the use of light-emitting diodes and fluorescent lamps in the same environment resulted in a change to HA production due to differences in leaf temperature (Matsuda et al. 2017b). In addition, HA production was improved by different factors such as photosynthetic photon flux density, planting density, and nitrogen concentration in the culture medium, except for carbon dioxide (Fujiuchi et al. 2014, 2017; Matsuda 2022; Matsuda et al. 2018, 2019).

However, since these conditions vary depending on the protein to be expressed and the plant species, it is essential to respond to individual conditions.

## Protein extraction, purification, and other costs and risks from plants

There are various methods of protein extraction from plants. The most commonly used methods for purification are bilayer partitioning using pH, ammonium sulfate precipitation, membrane separation, and protein tag purification (Coates et al. 2022). The process of removing plant-derived components, although not a big problem at the laboratory level, must be considered when producing recombinant proteins in large quantities. In particular, the removal of endogenous plant proteins, including ribulose-1,5-bisphosphate carboxylase/oxygenase (RuBisCO), which accounts for 50% of the soluble protein in leaf tissue, constitutes a crucial phase of the process in the purification of recombinant proteins (Holler et al. 2007). To remove an abundant protein such as RuBisCO in a variety of plants, polyethyleneimine (Zhang et al. 2013), ammonium sulfate (Loewen et al. 2013), trichloroacetic acid/acetone (Niu et al. 2018), and polyethylene glycol (Sehrawat et al. 2013) precipitation were well used. Recently, a freezing and thawing method in the purification of recombinant proteins from plants has been reported. According to this method, RuBisCO proteins could be reduced by about 10–20% without using specific reagents or equipment. However, damage to the expressed recombinant β-glucuronidase (GUS) protein was observed during freezing and thawing (Matsushima and Matsuo 2024). In addition, the cost of the freeze-thaw apparatus needs to be taken into account when production levels largely increase to commercial quantities. Since GUS proteins function by forming tetramers, the accuracy of protein purification by the freeze-thaw method may depend on the final molecular weight, conformation, and composition of the recombinant protein. The advantages of purification in freezing large quantities of leaf samples and the initial and running costs of the freezing equipment should be considered. Furthermore, recombinant proteins produced in plants are often purified as soluble proteins. Therefore, the use of centrifuges is not suitable for conditioning large numbers of leaf samples since this process involves significant costs, unlike laboratory experiments. In the early days, researchers tended to focus on efficient expression of recombinant proteins in plants and report less on isolation and purification; however, the purification part is the most costly and accounts for nearly 80% of all processes in protein production (Wilken and Nikolov 2012). Even if the cost of plant cultivation is low, it should be noted that the cost of the purification process is estimated to be comparable to other mammalian cell and microbial production approaches (Chen and Davis 2016). It would be necessary to use various chromatographic and other methods of mass purification.

WHO, in accordance with the World Health Organization Framework Convention on Tobacco Control (FCTC), which entered into force in 2005, places restrictions on trade with organizations associated with the tobacco industry (Article 5,3). In particular, the use of *Nicotiana benthamiana* as a host for the production of recombinant proteins for medical use in response to a pandemic is not considered to fall within the scope of the tobacco industry (Article 1d) or tobacco products (Article 1e) as defined by the FCTC, but it may need to be carefully considered, and we should be aware that the production of recombinant proteins has not only scientific challenges, but also economic and political issues that must be overcome.

## Conclusions and future perspectives

Recombinant proteins provided important breakthroughs in biomedical biotechnology. They are not only used in biomedical research but also for cosmetic, medical, therapeutic, and other industrial applications, and the demand for recombinant proteins is increasing.

Plants have advantages over bacteria, yeast, insects, mammalian cells, and other currently used methods, such as the safety of protein expression without endotoxin or amphixenosis, and the superior economic cost of scaling up production. It has been pointed out that there are ethical issues with the use of human-derived cells other than zoonosis (Duarte et al. 2023), which may limit their use if substitutes are made in the future. No major problems have been found with the expression system in plants, making this a promising technology for the future.

A few companies, described above, are undergoing regulatory approval processes to produce recombinant proteins transiently expressed in plants. So far, only COVIFENZ®, a SARS-CoV2 vaccine from Medicago Inc., has obtained full approval for human use, pending supply for pandemic use.

The recent discontinuation of sales of COVIFENZ® and the dissolution of that company came as a big shock to companies that produce useful recombinant products from plants. There are not only technical but also political problems (Duong and Vogel 2022) that must be overcome, but we will not be discouraged by this and will continue to produce useful foreign substances using plants.

## Abbreviations

CHO: Chinese hamster ovary cells
FCTC: Framework Convention on Tobacco Control
GILSP: Good Industrial Large-Scale Practice
GUS: β-glucuronidase
HA: hemagglutinin
HEK293: Human Embryonic Kidney cells 293
ROS: reactive oxygen species
RuBisCO: ribulose-1,5-bisphosphate carboxylase/oxygenase
